# Association of SIRI and NLPR with post-operative pneumonia in middle-aged and elderly patients after hip fracture surgery

**DOI:** 10.3389/fmed.2026.1861666

**Published:** 2026-07-01

**Authors:** Yan Tan, Fang Chai

**Affiliations:** 1 School of Public Health, Hangzhou Medical College, Hangzhou, Zhejiang, China; 2 Center for Rehabilitation Medicine, Department of Orthopedics, Zhejiang Provincial People's Hospital (Affiliated People's Hospital), Hangzhou Medical College, Hangzhou, Zhejiang, China

**Keywords:** hip fracture, middle-aged and elderly, NLPR, post-operative pneumonia, SIRI

## Abstract

**Background:**

Post-operative pneumonia (POP) is one of the most common complications following hip fracture surgery. The Systemic Inflammation Response Index (SIRI) and the Neutrophil-to-Lymphocyte-to-Platelet Ratio (NLPR) are novel inflammatory markers derived from peripheral blood cell counts. This study aims to evaluate the association of SIRI and NLPR at admission with hospital-acquired post-operative pneumonia in middle-aged and elderly patients after hip fracture surgery.

**Methods:**

A retrospective study was conducted with 452 patients aged 45 years or older who underwent hip fracture surgery. The Systemic Inflammation Response Index (SIRI) and the Neutrophil-to-Lymphocyte-to-Platelet Ratio (NLPR) were calculated based on peripheral blood cell counts (including neutrophil, monocyte, lymphocyte, and platelet counts) measured at admission. According to the occurrence of hospital-acquired post-operative pneumonia, patients were divided into a pneumonia group (*n* = 25) and a non-pneumonia group (*n* = 427). Multivariable logistic regression analysis was employed to evaluate the association of SIRI and NLPR with hospital-acquired post-operative pneumonia after hip fracture. Receiver operating characteristic (ROC) curve analysis was used to assess the performance of SIRI and NLPR for post-operative pneumonia and to determine the optimal cut-off values for each indicator. Additionally, restricted cubic spline analysis adjusted for covariates and subgroup analysis were performed.

**Results:**

A total of 452 patients were included in this study, of whom 25 (5.53%) developed post-operative pneumonia. Both SIRI and NLPR showed a positive association with the risk of pneumonia after hip fracture surgery. Both SIRI and NLPR showed moderate discriminative performance (AUC: 0.701 and 0.738, respectively). The difference between the two AUCs was not statistically significant (DeLong test, *P* = 0.247). An elevated NLPR above the optimal cut-off value of 2.879 was significantly associated with an increased incidence of POP (*OR* = 5.47, 95% CI: 1.95–20.8). Furthermore, additional restricted cubic spline and subgroup analyses supported the robustness of this finding.

**Conclusions:**

Both SIRI and NLPR are associated with the occurrence of hospital-acquired post-operative pneumonia in middle-aged and elderly patients with hip fracture. Both markers demonstrated moderate discriminative ability, with high negative predictive values, suggesting they may be useful as auxiliary screening tools for ruling out POP in low-risk patients. The numerically higher AUC of NLPR did not reach statistical significance compared with SIRI. However, given the low positive predictive value, it is more suitable for ruling out low-risk patients rather than definitively identifying those who will develop post-operative pneumonia. Its clinical utility as an early warning indicator requires further external validation.

## Introduction

With the accelerating pace of global population aging, hip fractures have emerged as an increasingly severe public health issue. Epidemiological studies indicate that by 2050, the global number of hip fracture patients is projected to increase to approximately 6.3 million (1). In China, the annual number of new hip fracture cases has already exceeded 1 million and continues to show a rising trend (2). As the most devastating type of osteoporotic fracture, hip fractures are associated with high rates of complications and mortality, posing a serious threat to the life and health of elderly patients (3, 4).

Post-operative pneumonia (POP) is the most common complication in hip fracture patients and a leading cause of post-operative mortality (5). Studies report that the incidence of POP in hip fracture patients ranges from 3 to 9%, underscoring the critical importance of early risk identification and proactive intervention for this complication (6–11).

In recent years, novel inflammatory markers derived from the complete blood cell count have been widely applied to predict various diseases and their outcomes (12–16). Among these, the Neutrophil-to- Lymphocyte-to-Platelet Ratio (NLPR) and the Systemic Inflammation Response Index (SIRI) are composite indices that integrate information from multiple immune cell lines, demonstrating superior predictive potential compared to traditional markers. NLPR, derived from the Neutrophil-to-Lymphocyte Ratio (NLR) by incorporating platelet count, serves to assess both inflammatory and coagulation pathways (17). It was initially established as an effective prognostic marker for patients with urosepsis (18). Currently, NLPR has been extensively utilized in research on infectious diseases (19, 20). Research has shown that in patients with COVID-19, the AUC of NLPR for predicting disease severity was significantly higher than that of NLR (21). Furthermore, a study on pediatric Mycoplasma pneumoniae pneumonia demonstrated that the predictive value of SIRI (AUC = 0.892) was superior to that of the Systemic Immune-inflammation Index (SII), NLR, and other dual-parameter indices (22). Collectively, this evidence suggests that composite indices like SIRI and NLPR, by reflecting the body's comprehensive pathological state, may offer advantages over single or dual-parameter markers.

However, although SIRI and NLPR have demonstrated promising application prospects in infectious diseases, their value in the context of post-operative complications following hip fracture, particularly in predicting post-operative pneumonia, has not been fully explored. Current research on post-operative pneumonia after hip fracture primarily relies on traditional inflammatory markers, such as the Neutrophil-to- Lymphocyte Ratio (NLR), Platelet-to-Lymphocyte Ratio (PLR), and Systemic Immune-inflammation Index (SII) (23). Although these markers have shown some predictive utility, they possess inherent limitations, creating a pressing need to identify more comprehensive and accurate predictive indicators.

Against this backdrop, this study aims to address this research gap. We intend to conduct a retrospective analysis of a cohort of middle-aged and elderly patients who underwent surgical treatment for hip fractures. By collecting pre-operative clinical data and laboratory parameters, we will systematically evaluate the association between pre-operative SIRI and NLPR levels and the occurrence of hospital-acquired post-operative pneumonia. Furthermore, we will employ statistical methods, including Receiver Operating Characteristic (ROC) curve analysis, to validate the predictive performance of SIRI and NLPR. The ultimate objective of this research is to explore whether SIRI and NLPR are associated with hospital-acquired post-operative pneumonia in hip fracture patients. If validated, these indices could provide new evidence-based medical support for the early identification of high-risk patients, the development of individualized intervention strategies, and ultimately, the improvement of patient outcomes.

## Materials and methods

This retrospective cohort study included 452 middle-aged and elderly patients (age ≥45 years) who underwent surgical treatment for hip fracture at Zhejiang Provincial People's Hospital between October 2018 and October 2022. The study protocol was reviewed and approved by the Institutional Review Board / Ethics Committee of Zhejiang Provincial People's Hospital. As the data used were retrospective and anonymized, the requirement for informed consent was waived. This study was conducted in accordance with the principles of the Declaration of Helsinki and reported following the STROBE (Strengthening the Reporting of Observational Studies in Epidemiology) guidelines.

**Figure 1:**
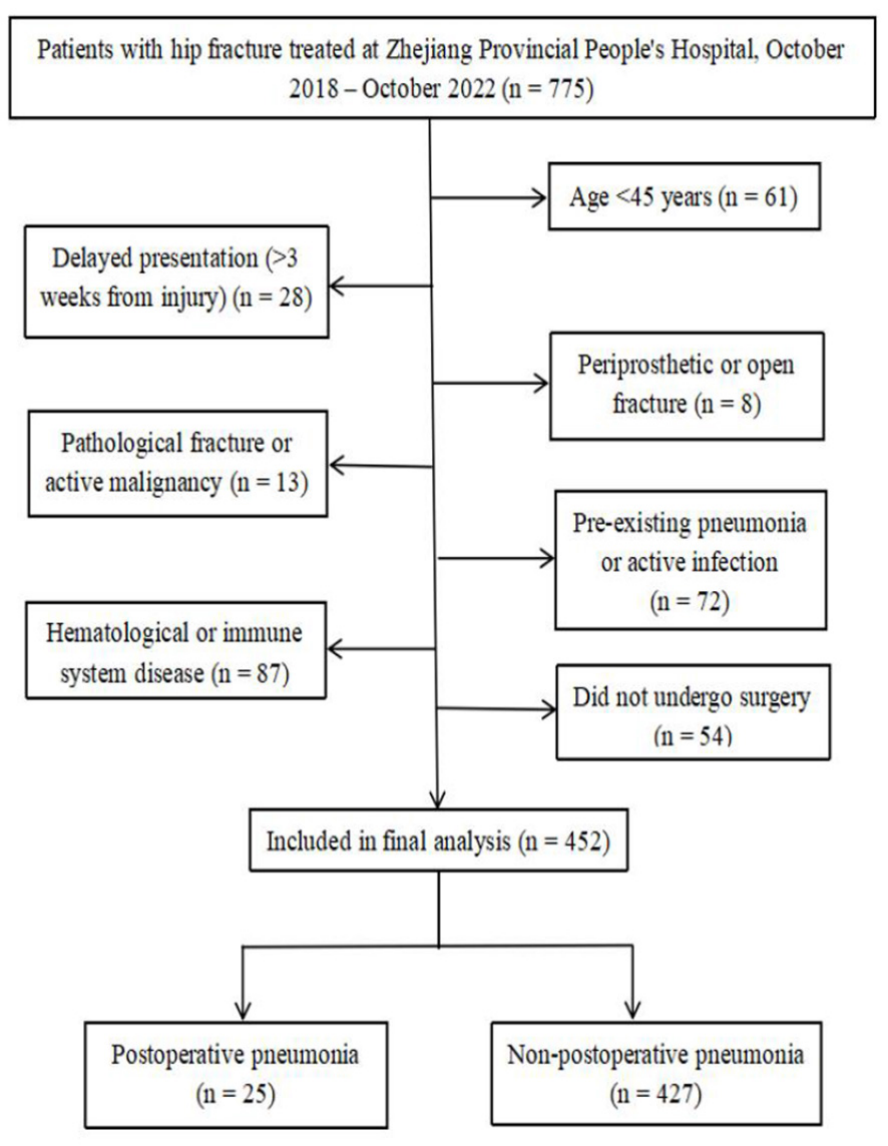
Patient flow diagram.

Patients were enrolled if they met the following criteria: (1) age ≥45 years (to include both middle-aged and older adults, as hip fracture incidence increases from middle age); and (2) diagnosis of hip fracture (including femoral neck fracture or intertrochanteric fracture) confirmed by radiographic examination. Patients were excluded based on the following criteria: (1) delayed presentation (time from injury to admission exceeding 3 weeks); (2) periprosthetic fracture; (3) open fracture; (4) pathological fracture due to underlying tumor; (5) active malignancy; (6) pre-existing pneumonia or other active infectious diseases confirmed before surgery; (7) comorbid hematological or immune system diseases, such as leukemia, rheumatoid arthritis, or systemic lupus erythematosus; or (8) did not undergo surgical intervention. A total of 452 participants were included in the final analysis. The participant inclusion flowchart is shown in Figure 1.

### Exposure

For all patients diagnosed with a hip fracture, the first peripheral blood cell count results following hospital admission were collected. These included the absolute counts of neutrophils (GR), lymphocytes (LY), monocytes (MO), and platelets (PLT). The Systemic Inflammation Response Index (SIRI) and the Neutrophil-to-Lymphocyte-to-Platelet Ratio (NLPR) were calculated using the following formulas: SIRI = (monocyte count × neutrophil count)/lymphocyte count, NLPR = (neutrophil count × 100)/(lymphocyte count × platelet count). This scaling factor ( × 100) was applied to facilitate clinical interpretation. The calculation methods were consistent with those described in previous studies (24, 25).

### Outcome

The diagnosis of post-operative hospital-acquired pneumonia (HAP) was established according to the guidelines outlined in the “Chinese Guidelines for the Diagnosis and Treatment of Hospital-Acquired Pneumonia and Ventilator-Associated Pneumonia in Adults (2018 Edition)” (26). The diagnostic criteria were as follows: (1) fever, with a body temperature >38.0 °C; (2) presence of clinical signs and symptoms such as purulent respiratory secretions; (3) white blood cell count >10 × 10^9^/L or < 4 × 10^9^/L; and (4) new or progressive infiltrates, consolidation, or ground-glass opacities observed on chest X-ray or computed tomography (CT). A confirmed diagnosis of HAP required meeting any two or more of the first three criteria (1–3) along with the radiological criterion (4), while simultaneously excluding other infectious diseases that could involve the lungs. The primary outcome of this study was the occurrence of HAP during the post-operative hospital stay. The observation period for this outcome commenced 24 h after surgery and lasted throughout the entire hospitalization period.

### Covariables

Patient clinical data were retrieved through the hospital's electronic medical record system. The collected information encompassed demographic characteristics, comorbidities, and surgery-related parameters. Demographic data included age and sex. Comorbidity data included a history of hypertension, heart disease, and the presence of any comorbidities. Surgery-related information included the fracture site (femoral neck fracture or intertrochanteric fracture), type of surgery (total hip arthroplasty, hemiarthroplasty, or internal fixation), time from fracture to surgery, duration of surgery, type of anesthesia (general or intraspinal), as well as the results from the first peripheral blood test after admission. This included the absolute counts of neutrophils (NEU), lymphocytes (Lym), platelets (PLT), and monocytes (MONO). All relevant parameters were explicitly documented and analyzed to ensure methodological rigor and reproducibility.

### Statistical analysis

All variables used in the primary and subgroup analyses were fully observed in the electronic medical record system. No missing data were present for any of the 452 included patients; therefore, neither imputation nor exclusion due to missingness was required, and the complete-case analytical sample comprised all 452 patients. Continuous variables are presented as mean ± standard deviation (SD) and were compared between groups using the independent samples *t*-test, provided they followed a normal distribution. Non-normally distributed continuous variables are expressed as median (interquartile range) and were compared using the Mann-Whitney U test. Categorical variables are presented as frequencies and percentages (%), and group comparisons were performed using the χ^2^ test or Fisher's exact test (when expected cell counts were < 5). Receiver operating characteristic (ROC) curve analysis was employed to evaluate the predictive performance of SIRI and NLPR for post-operative pneumonia. The area under the curve (AUC) and its 95% confidence interval were calculated. The difference between the AUCs of SIRI and NLPR was compared using the DeLong test for correlated ROC curves. Additionally, the significance of each AUC vs. 0.5 was assessed. The optimal cut-off value was determined, and the corresponding sensitivity, specificity, positive predictive value (PPV), and negative predictive value (NPV) were computed.

Given the low event rate (5.53%, *n* = 25), we followed the “one in ten” rule (10 events per covariate) to avoid overfitting. Guided by causal reasoning, we identified age and sex as the minimal sufficient adjustment set. Other covariates (hypertension, heart disease, comorbidities, fracture type, surgery method, time to surgery, operative time, anesthesia) were not included in the primary model because they may be intermediate variables or lack strong causal evidence; they are only examined in sensitivity analyses (Supplementary material 1). Due to the low event rate, Firth's penalized logistic regression (bias-reduced penalized maximum likelihood estimation) was used as the primary analytical method. Two models were constructed: Model 1 (unadjusted) and Model 2 (adjusted for age and sex). Bootstrap internal validation (1,000 resamples) with optimism correction was performed for Model 2 using the dichotomized SIRI and NLPR variables. The apparent AUC, optimism-corrected AUC, and 95% CI were reported.

To explore the dose-response relationship between SIRI and NLPR as continuous variables and the risk of post-operative pneumonia, restricted cubic spline (RCS) models with three knots were fitted based on the fully adjusted logistic regression model (Model 2), and the significance of non-linear associations was tested. Exploratory subgroup analyses were performed stratified by age (< 65 and ≥65 years), sex, comorbidities, fracture type, hypertension, and heart disease. Given the limited number of outcome events (*n* = 25), these analyses are underpowered and should be interpreted as hypothesis-generating. Interaction effects were assessed using the likelihood ratio test. All statistical analyses were conducted using R software (version 4.5.1). A two-sided *p*-value < 0.05 was considered statistically significant.

## Results

### Comparison of clinical characteristics

A total of 452 patients were included in this study, comprising 286 females (63.27%) and 166 males (36.73%). Among all participants, 25 (5.53%) developed post-operative pneumonia (POP) following hip fracture surgery. Compared to the non-POP group, the POP group had a higher prevalence of hypertension, heart disease, and other comorbidities, as well as a greater proportion of intertrochanteric fractures. SIRI and NLPR values were significantly elevated in the POP group. Neutrophil was significantly higher in the POP group, whereas platelet and lymphocyte counts were significantly lower (all *P* < 0.05). Detailed results are summarized in Table 1.

**Table 1:** Baseline characteristics of the 452 participants.

Characteristic	Non-POP *N* = 427^*a*^	POP *N* = 25^*a*^	*p*-value^*b*^
Age (years)	77 (66, 84)	76 (72, 85)	0.373
Sex			
Male	155 (36%)	11 (44%)	0.438
Female	272 (64%)	14 (56%)	
Hypertension			
Yes	201 (47%)	17 (68%)	0.042
No	226 (53%)	8 (32%)	
Heart disease			
Yes	64 (15%)	8 (32%)	0.043
No	363 (85%)	17 (68%)	
Comorbidity			
Yes	249 (58%)	20 (80%)	0.032
No	178 (42%)	5 (20%)	
Fracture type			
Femoral neck fracture	265 (62%)	10 (40%)	0.028
Intertrochanteric fracture	162 (38%)	15 (60%)	
Operation method			
Total Hip Arthroplasty	94 (22%)	2 (8.0%)	0.242
Hemiarthroplasty	108 (25%)	8 (32%)	
Internal fixation	225 (53%)	15 (60%)	
Time from fracture to surgery (days)			
0–2	137 (32%)	6 (24%)	0.733
3–7	266 (62%)	18 (72%)	
>7	24 (5.6%)	1 (4.0%)	
Intraoperative time (min)			
< 90	231 (54%)	15 (60%)	0.887
90–119	117 (27%)	6 (24%)	
≥120	79 (19%)	4 (16%)	
Anesthesia method			
General anesthesia	290 (68%)	17 (68%)	0.993
Intraspinal anesthesia	137 (32%)	8 (32%)	
Platelet count ( × 10^9^/L)	182 (148, 225)	154 (145, 198)	0.028
Neutrophil count ( × 10^9^/L)	6.11 (4.80, 7.80)	7.70 (5.06, 9.80)	0.018
Lymphocyte count ( × 10^9^/L)	1.20 (0.90, 1.43)	1.00 (0.80, 1.14)	0.017
Monocyte count ( × 10^9^/L)	0.50 (0.39, 0.60)	0.50 (0.40, 0.70)	0.339
SIRI	2.50 (1.70, 4.00)	4.69 (2.61, 5.37)	< 0.001
NLPR	2.91 (1.97, 4.56)	5.76 (3.08, 6.88)	< 0.001

^*a*^Median (Q1, Q3); *n* (%). ^*b*^Wilcoxon rank sum test; Pearson's Chi-squared test; Fisher's exact test.

### Inflammatory biomarkers of the study population

**Figure 2:**
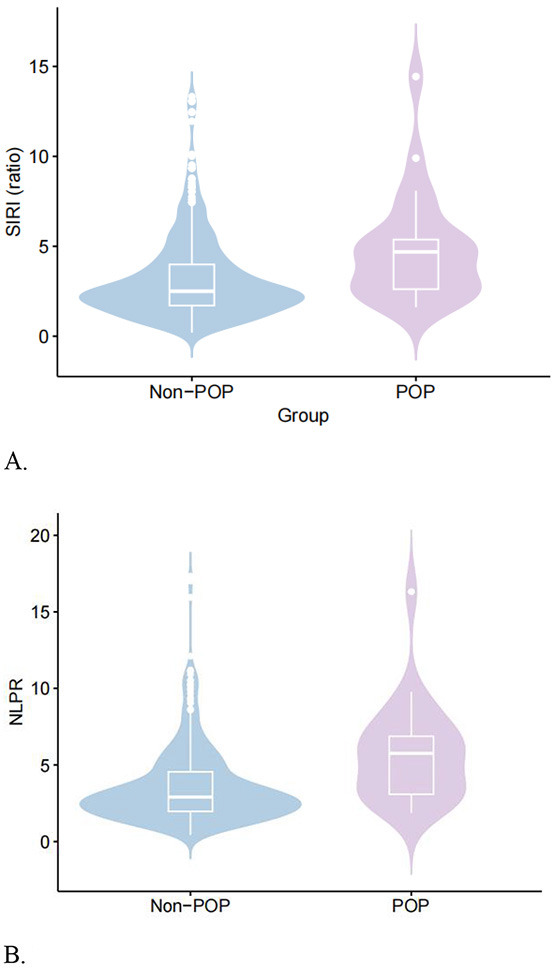
Violin plots of SIRI **(A)** and NLPR **(B)** in the non-POP and POP groups. **(A)** The SIRI of the POP group was higher than that of the non-POP group (*P* < 0.001); **(B)** The NLPR of the POP group was higher than that of the non-POP group (*P* < 0.001).

**Figure 3:**
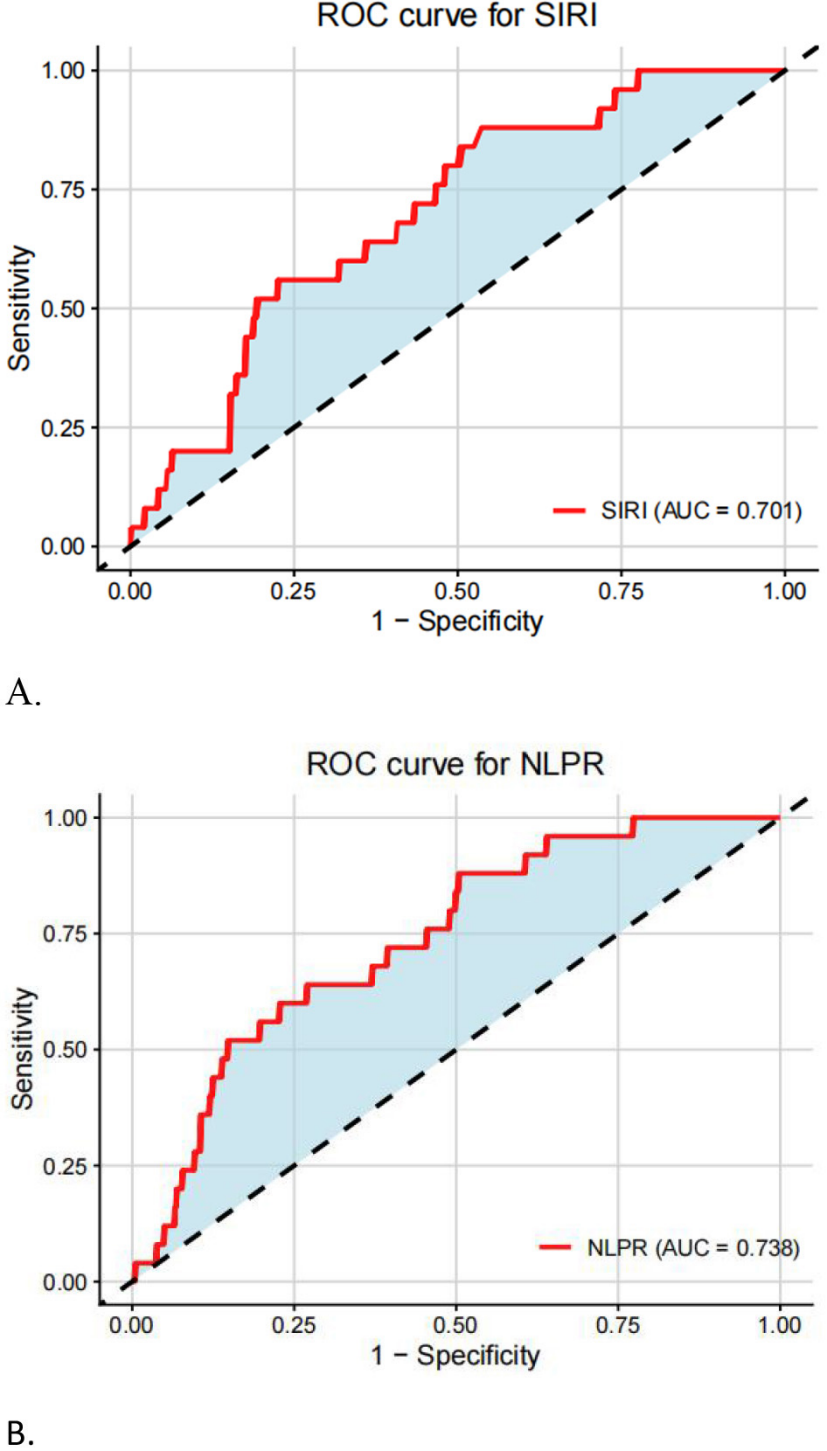
The ROC curves of SIRI and NLPR in predicting the occurrence of POP. **(A)** ROC curve for SIRI; **(B)** ROC curve for NLPR.

Violin plots demonstrated significantly higher levels of both SIRI (*P* < 0.001) and NLPR (*P* < 0.001) in the POP group compared to the non-POP group (Figure 2). Receiver operating characteristic (ROC) analysis was employed to evaluate the predictive performance of these biomarkers for hospital-acquired pneumonia following hip fracture surgery (Figure 3). The ROC curve analysis determined the optimal cut-off values for the continuous inflammatory biomarkers as 2.394 for SIRI and 2.879 for NLPR (Table 2). The AUCs were 0.738 (95% CI: 0.647–0.828) for NLPR and 0.701 (95% CI: 0.606–0.795) for SIRI. Direct comparison using the DeLong test for correlated ROC curves showed that the difference between the two AUCs was not statistically significant (*P* = 0.247). However, as shown in Table 2, the positive predictive values were low (8.8% for SIRI and 9.3% for NLPR), while the negative predictive values were high (both >98%). This indicates that these markers are more useful for ruling out POP in patients with low values than for confidently predicting who will develop POP.

**Table 2:** Assessment of the characteristic parameters of each biomarker.

Variables	Cutoff point	AUC (95% CI)	SEN (%, 95% CI)	SPE (%, 95% CI)	PPV (%, 95% CI)	NPV (%, 95% CI)	*p*-value
SIRI	2.394	0.701 (0.606, 0.795)	88 (70, 95.8)	46.4 (41.7, 51.1)	8.8 (5.9, 12.9)	98.5 (95.7, 99.5)	< 0.001
NLPR	2.879	0.738 (0.647, 0.828)	88 (70, 95.8)	49.6 (44.9, 54.4)	9.3 (6.2, 13.7)	98.6 (96, 99.5)	< 0.001

CI, confidence interval; AUC, the area under the curve; SEN, sensitivity; SPE, specificity; PPV, positive predictive value; NPV, negative predictive value. The *p*-values shown are for each AUC compared to 0.5 (indicating discrimination better than chance). The difference between the two AUCs was not statistically significant (DeLong test, *P* = 0.247). SIRI = (monocyte count × neutrophil count) / lymphocyte count. NLPR = (neutrophil count × 100) / (lymphocyte count × platelet count).

### Firth's penalized logistic regression

Firth's penalized logistic regression showed that both SIRI and NLPR were significantly associated with post-operative pneumonia (Table 3). After adjustment for age and sex, each one-unit increase in SIRI was associated with an *OR* of 1.21 (95% CI: 1.06–1.37, *P* = 0.005); for NLPR, *OR* = 1.22 (95% CI: 1.09–1.36, *P* = 0.001). Using the optimal cut-offs, high SIRI (≥ 2.394) had an *OR* of 5.27 (95% CI: 1.88–20.1, *P* < 0.001); high NLPR (≥ 2.879) had an *OR* of 5.98 (95% CI: 2.13–22.8, *P* < 0.001).

**Table 3:** Multivariable Firth's penalized logistic regression analysis of the associations of SIRI and NLPR with post-operative pneumonia (POP).

Biomarker	Group	Model 1	*P*	Model 2	*P*
SIRI	Continuous	1.22 (1.08, 1.38)	0.003	1.21 (1.06, 1.37)	0.005
	Best cutoff		< 0.001		< 0.001
	< 2.394	1 [Reference]		1 [Reference]	< 0.001
	≥ 2.394	5.56 (2.00, 21.1)		5.27 (1.88, 20.1)	
NLPR	Continuous	1.23 (1.10, 1.37)	< 0.001	1.22 (1.09, 1.36)	0.001
	Best cutoff		< 0.001		< 0.001
	< 2.879	1 [Reference]	< 0.001	1 [Reference]	< 0.001
	≥2.879	6.34 (2.28, 24.0)		5.98 (2.13, 22.8)	

Model 1 adjusted for: none; Model 2 adjusted for age and sex.

### Internal validation

Bootstrap internal validation with 1,000 resamples was performed on Model 2. For the dichotomized NLPR (≥2.879), the apparent AUC was 0.709, with an optimism-corrected AUC of 0.695 (95% CI: 0.636–0.729; optimism = 0.014). For the dichotomized SIRI (≥2.394), the apparent AUC was 0.702, with a corrected AUC of 0.686 (95% CI: 0.634–0.729; optimism = 0.016). The small optimism values indicated minimal overfitting and good internal validity.

Sensitivity analyses using conventional logistic regression and fully adjusted Firth's penalized logistic regression (Model 3) are presented in Supplementary material 1.

### Restricted cubic spline (RCS)

**Figure 4:**
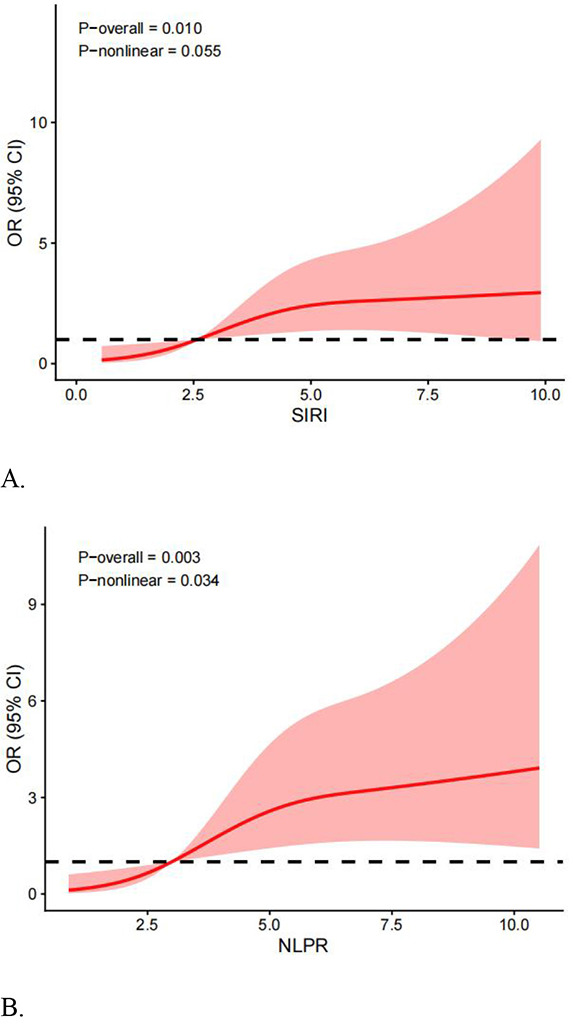
Restricted cubic spline analyses of SIRI and NLPR in relation to post-operative pneumonia. **(A)** Restricted cubic spline (RCS) analysis of SIRI based on multivariable Model 2; **(B)** Restricted cubic spline (RCS) analysis of NLPR based on multivariable Model 2.

To further investigate the dose-response relationship between SIRI, NLPR, and the risk of post-operative pneumonia, we performed restricted cubic spline (RCS) regression with three knots, adjusted for age and sex (Model 2). As shown in Figure 4, the RCS analysis revealed a significant non-linear relationship for NLPR (*P*-non-linear = 0.034), indicating that the risk of POP does not increase linearly with rising NLPR levels. In contrast, the association for SIRI was linear (*P*-non-linear > 0.05). These results suggest that NLPR may have a threshold or saturation effect on POP risk.

### Subgroup analysis

**Figure 5:**
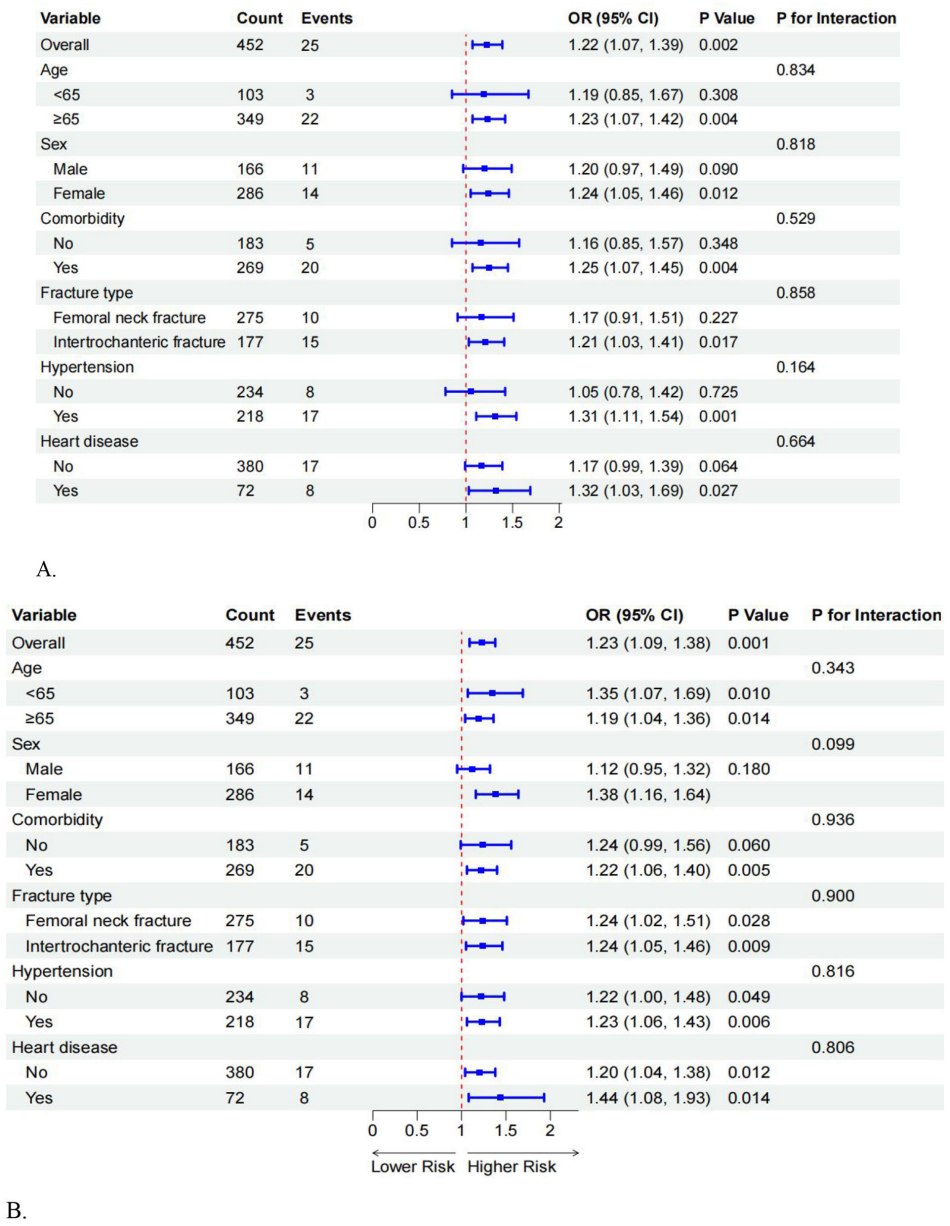
Subgroup analysis. **(A)** Subgroup analysis of the association between SIRI and post-operative pneumonia; **(B)** Subgroup analysis of the association between NLPR and post-operative pneumonia.

Exploratory subgroup analyses were performed for age, sex, comorbidity, fracture type, hypertension, and heart disease (Figure 5). Due to the low overall event rate (5.53%, *n* = 25), these analyses are underpowered. Interaction tests were non-significant for all subgroups (all *P* for interaction > 0.05). However, non-significant interaction tests do not prove the absence of effect modification, and these results should be interpreted with caution. The number of events in each subgroup is displayed in Figure 5.

## Discussion

Post-operative pneumonia (POP) following hip fracture surgery significantly increases patient mortality, prolongs hospital stays, and elevates healthcare costs (27, 28), representing a pressing clinical Challenge. This study found that both the Systemic Inflammation Response Index (SIRI) and the Neutrophil-to-Lymphocyte-to-Platelet Ratio (NLPR), measured at admission, were significantly associated with hospital-acquired POP in middle-aged and elderly hip fracture patients. NLPR showed a numerically higher AUC (0.738) than SIRI (0.701), but the difference was not statistically significant (DeLong test, *P* = 0.247). Nonetheless, both markers demonstrated moderate discriminative ability, and the optimal cut-off value of 2.879 for NLPR may serve as a practical threshold for initial risk stratification. Given the high negative predictive value (98.6%), NLPR is particularly useful for excluding low-risk patients, thereby allowing clinicians to focus monitoring resources on higher-risk individuals. Nevertheless, the low positive predictive value (9.3%) indicates that a positive result alone does not confirm impending pneumonia, and clinical judgment remains essential. The low specificity (49.6%) further limits the ability to confirm POP, reinforcing that NLPR is more suitable for ruling out than ruling in the complication. Furthermore, the restricted cubic spline (RCS) analyses revealed a significant non-linear relationship for NLPR *(P*-non-linear = 0.034) and a linear relationship for SIRI (*P*-non-linear > 0.05), indicating distinct dose-response patterns. Together with the robustness confirmed by subgroup analyses, these findings suggest potential application value of these biomarkers, particularly NLPR, in POP risk stratification.

In recent years, inflammatory biomarkers derived from routine peripheral blood parameters have gained increasing attention for prognostic assessment in various diseases due to their accessibility, cost-effectiveness, and reproducibility (29, 30). Although the pathogenesis of POP is multifactorial, perioperative systemic inflammation is a key pre-disposing factor (31). SIRI, integrating neutrophil, monocyte, and lymphocyte counts, concurrently reflects pro-inflammatory activation and lymphocyte-related immunosuppression (32). Our findings align with studies in other conditions, such as stroke-associated pneumonia (33), confirming SIRI's predictive value for POP and extending its potential clinical applicability.

This study is the first to systematically evaluate and compare the predictive value of NLPR and SIRI for post-operative pneumonia specifically in a hip fracture population, offering new tools for clinical risk assessment. Compared to previous studies focusing on the association between Neutrophil-to-Lymphocyte Ratio (NLR) and POP after hip fracture (reported AUC = 0.648) (23), both NLPR (AUC = 0.738) and SIRI (AUC = 0.701) showed improved discriminative performance, although the difference between NLPR and SIRI was not statistically significant. This advantage may stem from NLPR's unique capacity to capture the interplay between inflammation, immunity, and coagulation in the pathogenesis of POP (34). Hip fracture and surgical trauma trigger a neutrophil surge (promoting inflammation), lymphopenia (impairing immunity), and platelet activation (35). Activated platelets not only contribute to a prothrombotic state but also release inflammatory mediators that can exacerbate lung injury (36). As a novel index integrating these three pathways, NLPR provides a more comprehensive risk assessment than markers lacking platelet counts, such as NLR or SIRI. It should be noted, however, that the difference between NLPR and SIRI did not reach statistical significance in our study; therefore, the theoretical advantages of NLPR over SIRI remain speculative and require further investigation.

Our RCS analysis revealed a linear relationship for SIRI (*P*-non-linear > 0.05) but a significant non-linear relationship for NLPR (*P*-non-linear = 0.034), suggesting that the association between NLPR and POP risk is not simply linear. Exploratory subgroup analyses suggested consistent directions of association for SIRI and NLPR with POP across most strata of age, sex, comorbidities, fracture type, hypertension, and heart disease (Figure 5). However, the small number of events (*n* = 25) substantially limits statistical power and precludes definitive conclusions regarding effect modification. Interaction tests were non-significant for all subgroups (all *P* for interaction > 0.05), but these findings should be interpreted as hypothesis-generating rather than confirmatory. Future adequately powered studies are warranted to robustly evaluate potential effect modification by these factors.

It is important to acknowledge that although NLPR demonstrated acceptable discrimination (AUC = 0.738), calibration analysis and decision curve analysis (DCA) were not performed in this study. These analyses are essential for evaluating the clinical utility of a biomarker, particularly in scenarios with low event rates. Without DCA, we cannot quantify the net benefit of using NLPR to guide clinical decisions. Furthermore, our findings are based on a single-center retrospective cohort, and external validation in independent, multi-center populations is mandatory before NLPR can be considered for routine clinical application.

However, several limitations must be acknowledged. First, the single-center, retrospective design inherently carries a risk of unmeasured confounding and limits the generalizability of the results. The relatively low incidence of POP in our cohort (5.53%, *n* = 25), although within the reported range, resulted in wide confidence intervals in regression models. Although we employed Firth's penalized logistic regression to provide more robust estimates, future studies with larger sample sizes are needed to obtain more precise effect estimates. Second, as with all observational studies, we can only establish associations, not causality. Third, biomarkers were measured only once at admission; their dynamic changes during the perioperative period might offer additional prognostic information but were not captured in this study. Finally, external validation in independent, larger, multi-center cohorts is necessary to confirm our findings.

## Conclusions

This study found that pre-operative SIRI and NLPR are independently associated with hospital-acquired pneumonia after hip fracture surgery. Both markers showed moderate discriminative ability (AUC 0.701–0.738) and high negative predictive values (>98%), indicating they may be useful for ruling out POP in low-risk patients. The optimal cut-off value of 2.879 for NLPR may assist in initial risk stratification, but the difference in AUC between NLPR and SIRI was not statistically significant (DeLong test, *P* = 0.247). Given the modest positive predictive value and lack of calibration/clinical utility analyses, these biomarkers should not be used in isolation to confirm high risk. Their definitive clinical utility requires prospective, multi-center validation incorporating decision curve analysis.

## Data Availability

The raw data supporting the conclusions of this article will be made available by the authors, without undue reservation.
