# Supplementary material for: Association of SIRI and NLPR with post-operative pneumonia in middle-aged and elderly patients after hip fracture surgery
Source: Front Med (Lausanne). 2026 Jul 1;13:1861666. doi: 10.3389/fmed.2026.1861666 (PMC13368979; doi:10.3389/fmed.2026.1861666)
Supplement: Supplementary file 1 [file Table_1.DOCX]

**Supplementary Table 1. Multivariable Firth’s logistic regression analysis (Model 3) for the associations of SIRI and NLPR with postoperative pneumonia (POP).**

| **Biomarker** | **Group** | Model 3 | *P* |
| --- | --- | --- | --- |
| **SIRI** | **Continuous** | 1.19(1.04, 1.36) | 0.012 |
|  | **Best cutoff** |  | 0.002 |
|  | <2.394 | 1 [Reference] |  |
|  | ≥ 2.394 | 4.69 (1.67, 17.9) |  |
| **NLPR** | **Continuous** | 1.21 (1.07, 1.36) | 0.003 |
|  | **Best cutoff** |  | <0.001 |
|  | <2.879 | 1 [Reference] |  |
|  | ≥2.879 | 5.47 (1.95, 20.8) |  |

Model 3 adjusted for age, sex, hypertension, heart disease, comorbidity, fracture type, surgery method, time from fracture to surgery (days), intraoperative time (min) and anesthesia method.

**Supplementary Table 2. Multivariable logistic regression analysis of the associations of SIRI and NLPR with postoperative pneumonia (POP).**

| **Biomarker** | **Group** | Model 1 | *P* | Model 2 | *P* | Model 3 | *P* |
| --- | --- | --- | --- | --- | --- | --- | --- |
| **SIRI** | **Continuous** | 1.22 (1.07, 1.38) | 0.002 | 1.21 (1.06, 1.37) | 0.004 | 1.21 (1.05, 1.38) | 0.008 |
|  | **Best cutoff** |  | 0.003 |  | 0.004 |  | 0.006 |
|  | <2.394 | 1 [Reference] |  | 1 [Reference] |  | 1 [Reference] |  |
|  | ≥ 2.394 | 6.34 (2.16, 27.1) |  | 6.06 (2.04, 26.0) |  | 5.74 (1.89, 25.0) |  |
| **NLPR** | **Continuous** | 1.23 (1.10, 1.38) | <0.001 | 1.22 (1.09, 1.37) | <0.001 | 1.23 (1.08, 1.40) | 0.001 |
|  | **Best cutoff** |  | 0.001 |  | 0.002 |  | 0.003 |
|  | <2.879 | 1 [Reference] |  | 1 [Reference] |  | 1 [Reference] |  |
|  | ≥2.879 | 7.23 (2.46, 30.9) |  | 6.86 (2.31, 29.5) |  | 6.81 (2.24, 29.7) |  |

Model 1 adjusted for: none;

Model 2 adjusted for age and sex;

Model 3 adjusted for age, sex, hypertension, heart disease, comorbidity, fracture type, surgery method, time from fracture to surgery (days), intraoperative time (min) and anesthesia method.
